# Diagnostic accuracy of gastrointestinal ultrasound in predicting enteral feeding intolerance: a systematic review and meta-analysis

**DOI:** 10.3389/fnut.2026.1767700

**Published:** 2026-04-20

**Authors:** Shiqi Jia, Yao Yin, Cong Wang, Shanshan Liu, Yan Jiang

**Affiliations:** 1 Department of Neurosurgery, West China Hospital, Sichuan university/West China School of Nursing, Sichuan University, Chengdu, China; 2 Evidence-Based Nursing Center, West China Hospital, Sichuan University, Chengdu, China; 3 Sichuan Provincial Engineering Research Center of Medical Nursing Equipment and Materials, Chengdu, China; 4 Department of Nursing, West China Hospital, Sichuan University/West China School of Nursing, Sichuan University, Chengdu, China

**Keywords:** diagnostic accuracy, enteral feeding intolerance, gastrointestinal ultrasound, meta-analysis, predict

## Abstract

**Background:**

Gastrointestinal ultrasonography (GI-US) has significant potential for predicting enteral nutrition intolerance (ENFI) in critically ill adults. However, current evidence is fragmented, with substantial variability in predictors, diagnostic thresholds, acquisition protocols, and a lack of cross-indicator comparisons. These inconsistencies hinder evidence synthesis and the standardization of clinical practice.

**Objective:**

This study aimed to systematically synthesize and evaluate the diagnostic performance of GI-US predictors for ENFI, identifying core predictors that balance accuracy with clinical feasibility.

**Methods:**

Comprehensive searches were conducted in PubMed, Web of Science, Embase, CINAHL, the Cochrane Library, CNKI, Wanfang, and SinoMed through September 12, 2024. Two independent reviewers screened studies, extracted data, and assessed quality using the QUADAS-2 tool. Diagnostic accuracy was analyzed using a bivariate random-effects model to generate summary estimates of sensitivity, specificity, likelihood ratios, diagnostic odds ratios, and SROC-AUCs, all with 95% confidence and prediction intervals. Threshold effects, heterogeneity, sensitivity analyses, publication bias (via Deeks’ test), and clinical utility (via Fagan nomograms) were comprehensively evaluated.

**Results:**

The analysis included 16 studies with 1,261 participants, evaluating ten single predictors, one composite score, and two multivariable models. Seven studies (*n* = 502) on gastric antrum cross-sectional area (GCSA) were suitable for meta-analysis, demonstrating good diagnostic accuracy (SROC-AUC 0.86, 95% CI 0.76–0.89; sensitivity 0.82, 95% CI 0.76–0.88; specificity 0.77, 95% CI 0.72–0.81), with no significant heterogeneity or publication bias. At a pretest probability of 42.91%, a positive GCSA result increased the posttest probability of ENFI to 72.51%, while a negative result decreased it to 14.74%. Other single predictors varied in accuracy, whereas composite scores and multivariable models achieved higher AUCs (up to 0.95) but were supported by limited evidence.

**Conclusion:**

Current GI-US predictors can be categorized into gastric, intestinal, and superior mesenteric artery parameters. GCSA is supported by strong evidence for quantitative synthesis, demonstrating reliable diagnostic accuracy and bedside feasibility. Other predictors and emerging models show promise but require validation. Large, standardized multicenter studies are needed to validate multidomain predictors, harmonize measurement protocols, and develop integrated frameworks for effective ENFI management.

**Systematic review registration:**

PROSPERO, Identifier CRD420251019447.

## 1 Introduction

Nutritional support is a fundamental component of care for critically ill patients. Enteral nutrition (EN) preserves gastrointestinal mucosal integrity, reduces infection and mortality rates, and shortens the hospital stay (1). Accordingly, the American Society for Parenteral and Enteral Nutrition (ASPEN) and the European Society for Parenteral and Enteral Nutrition (ESPEN) recommend EN as the first-line nutritional strategy for adults in intensive care units (ICUs) (2, 3). However, enteral feeding intolerance (ENFI), also referred to as feeding intolerance (FI), is characterized by increased gastric residual volume, vomiting, diarrhea, and other gastrointestinal manifestations (4). It affects approximately 30.5–65.7% of ICU patients receiving EN and frequently necessitates the interruption of feeding, which may lead to undernutrition and an increased risk of complications (5). The evidence indicates that early prediction and proactive intervention for FI, rather than reactive management, can improve patient outcomes (6).

FI reflects disturbances in gastrointestinal motility and mucosal function, creating the need for a monitoring tool capable of providing quantitative, real-time, dynamic, and structural information. Existing assessment methods have notable limitations. Symptom scoring depends on patient report and assessor judgment, limiting its utility in sedated or mechanically ventilated patients. Manual measurement of gastric residual volume (GRV), although simple, disrupts feeding. Imaging modalities such as X-ray, computed tomography (CT), and scintigraphy provide anatomical and emptying assessments but require patient transport and exposure to ionizing radiation. Antroduodenal manometry is invasive and technically complex; breath tests provide delayed results and are susceptible to systemic interference; and biochemical and hormonal assays are neither immediate nor suitable for bedside use.

Gastrointestinal ultrasound (GI-US) offers a solution that meets ICU requirements for “quantitative, reproducible, bedside, and real-time” assessment. GI-US is noninvasive, radiation free, and capable of bedside evaluation of multiple predictors, such as the gastric antrum cross-sectional area (GCSA), gastric wall thickness, and peristaltic frequency. In addition to informing routine clinical management (e.g., adjusting feeding rates or initiating prokinetics), GI-US predictors have demonstrated strong correlations with FI onset and can provide early warning signals hours to days before symptoms emerge, thereby shifting FI management from reactive to preventive.

Prospective observational studies support the utility of GI-US in proactive FI management. For example, El Khoury et al. (*n* = 44) reported a significantly greater median GCSA in patients who developed FI than in controls (970 mm^2^ vs. 553 mm^2^; *p* < 0.001), with an area under the curve (AUC) of 0.86, a sensitivity of 0.91, and a specificity of 0.81 (7). Yu et al. (*n* = 60) similarly reported elevated GCSAs and acute gastrointestinal injury ultrasonography (AGIUS) scores in FI patients (*p* < 0.05); the AUC for predicting FI within 1 week was 0.763 (sensitivity 86.0%, specificity 79.4%), whereas the AGIUS achieved an AUC of 0.827 (sensitivity 87.7%, specificity 82.4%) (8). Other predictors—including the GRV (9), peak superior mesenteric artery (SMA) velocity (10), and intestinal diameter and wall thickness (11)—have also demonstrated predictive potential.

Despite the increasing volume of research examining various GI-US parameters for predicting ENFI, the current body of evidence remains significantly constrained. First, the majority of studies have focused on exploring or validating individual parameters or small subsets of predictors without systematically synthesizing the diagnostic accuracies of diverse GI-US predictors. This has led to a lack of a coherent parameter landscape and a clear understanding of their relative performance. Second, considerable variability in diagnostic thresholds, measurement conditions, and operational techniques across studies hinders the development of standardized criteria and consensus recommendations. Third, direct comparisons of predictors are infrequent, and no study has definitively identified the most informative individual parameter or optimal combination model, limiting progress toward precision prediction tools. Furthermore, the clinical feasibility and bedside applicability of GI-US predictors have not been fully assessed, limiting the development of implementable pathways for routine clinical practice. Additionally, the existing evidence stems primarily from single-center, small-sample, and nonstandardized studies, which lack a robust multicenter evidence base. This restricts the standardized application of GI-US in ENFI prediction and hampers the development of clinical guidelines.

Consequently, there is an urgent need for a systematic approach that encompasses the full spectrum of GI-US predictors, establishes a clear parameter profile, quantifies diagnostic performance, and assesses clinical feasibility across various care settings. This study conducts a systematic review and meta-analysis to consolidate the diagnostic accuracy of all GI-US predictors documented in the literature and identify key parameters that demonstrate both robust performance and high clinical feasibility. The aim is to provide a solid evidence base for the early identification and accurate prediction of ENFI, ultimately informing the development and standardization of future multimodal prediction models.

## 2 Methods

This systematic review was reported in accordance with the Preferred Reporting Items for Systematic Reviews and Meta-Analyses of Diagnostic Test Accuracy (PRISMA-DTA) guidelines (12). The protocol was prospectively registered in the International Prospective Register of Systematic Reviews (PROSPERO; registration no. CRD420251019447).

### 2.1 Search strategy

Two independent reviewers systematically searched PubMed, Embase, Web of Science, CINAHL (via EBSCO), the Cochrane Library, China National Knowledge Infrastructure (CNKI), Wanfang Data, and SinoMed from database inception to 12 September 2024. The search strategies followed the PIRD framework, combining Medical Subject Headings (MeSH) and free-text terms.

The key components were as follows: population (P): “Enteral Nutrition” [MeSH]; index test (I): “Ultrasonography” [MeSH] OR “Ultrasonics” [MeSH]; and target condition (D): “feeding intolerance,” “gastric residual volume,” “energy intake,” and gastrointestinal symptoms such as “vomiting,” “abdominal distension,” and “diarrhea.” Because no universally accepted definition of FI exists, we used a broad range of terms to maximize retrieval. The complete strategies are provided in Supplementary Table 1. The reference lists of the included articles were manually screened, and targeted online searches were performed. Disagreements were resolved through discussion or by consulting a third reviewer.

### 2.2 Study selection

Titles and abstracts were screened independently by two reviewers, followed by full-text assessment via predefined inclusion and exclusion criteria. Discrepancies were resolved by consensus.

#### 2.2.1 Inclusion criteria

Studies were eligible if they met all the following criteria:

1. Participants: Adults (≥18 years) receiving or scheduled to receive EN, regardless of diagnosis; concurrent nutritional modalities permitted.
2. Reference standard: FI was considered present if any one of the following criteria was met: (a) FI was reported as an outcome with clearly specified diagnostic criteria; (b) synonymous terms were used (e.g., “gastrointestinal intolerance,” “enteral nutrition failure,” “gastrointestinal dysfunction”); or (c) the diagnostic definition included at least one of the following features: high GRV, inadequate EN delivery, or gastrointestinal symptoms (e.g., bloating, diarrhea, vomiting, constipation, reflux, aspiration, abdominal discomfort, and abnormal bowel sounds) (13).
3. Index tests: Assessment of at least one GI-US predictor at any time point (prefeeding, during feeding, or continuously).
4. Diagnostic accuracy outcomes: At least one sensitivity, specificity, positive likelihood ratio (PLR), negative likelihood ratio (NLR), diagnostic odds ratio (DOR), or AUC was used.
5. Study design: Prospective or retrospective cohort studies.

#### 2.2.2 Exclusion criteria

Studies were excluded on the basis of the following criteria: (1) failure to report ultrasound-based accuracy metrics separately; (2) availability limited to conference abstracts; (3) availability of data from the same cohort, with preference given to the earliest or most comprehensive report; (4) inability to retrieve the full text despite attempts to contact the authors; (5) publication in languages other than English or Chinese, as the majority of relevant evidence in this field is published in these languages; and (6) employment of study designs unsuitable for evaluating diagnostic or predictive accuracy, such as case–control studies, case series, or other single-arm descriptive designs; studies where GI-US was conducted only after the occurrence of FI; studies that included GI-US findings as part of the FI reference standard; or studies where GI-US and FI were not assessed as paired measures within the same EN cohort or where the temporal relationship between GI-US measurement and FI determination was unclear or inappropriate.

#### 2.2.3 Meta-analysis eligibility

All eligible studies were qualitatively summarized. Quantitative meta-analysis was performed only for GI-US predictors that were assessed in ≥5 studies for which complete or reconstructible 2 × 2 contingency tables were available (14). Furthermore, studies had to be conceptually comparable with respect to the definition of the predictor and the reference standard for FI to be included in any pooled analysis.

### 2.3 Data extraction

Using a piloted, standardized form, two reviewers independently extracted the following data: (1) Study characteristics: first author year, country, funding, study design, number of centers, sample size, setting, GI–US parameters reported and FI–directed interventions. (2) Participant characteristics: age (mean ± SD) and sex distribution. (3) Acquisition and evaluation of GI-US predictors: specific predictors, calculation formula, timing of measurement, assessor qualifications, FI diagnostic criteria, and assessment period. (4) Diagnostic accuracy data: 2 × 2 table values, sensitivity, specificity, PLR, NLR, DOR, and AUC.

When complete 2 × 2 contingency tables were not reported, a predefined hierarchical approach was applied. Efforts were made to contact the original authors to obtain missing information. If these attempts were unsuccessful, the 2 × 2 data were reconstructed, where feasible, using sensitivity, specificity, prevalence, or other diagnostic metrics provided in the article. For studies that reported only ROC curves, the optimal threshold and its corresponding sensitivity and specificity were extracted by digitizing the curve (using the GetData Graph Digitizer v2.24). When multiple potentially eligible data points for the same predictor were provided by a single study, the following selection principles were applied for the primary analysis: (1) if a study-defined diagnostic threshold was available, data corresponding to this threshold were prioritized; (2) in the absence of a predefined threshold, data obtained under measurement conditions most consistent with routine clinical practice or the most commonly used assessment method were preferentially selected; and (3) for data not conforming to the above principles, the dataset most consistent with the majority of included studies was used for pooling. The remaining data were included in the sensitivity analyses. Throughout the process, discrepancies were resolved through discussion or adjudication by a third reviewer.

### 2.4 Quality assessment

The methodological quality was independently assessed by two reviewers via the revised Quality Assessment of Diagnostic Accuracy Studies-2 (QUADAS-2) tool (15), which evaluates four domains: patient selection, index test, reference standard, and flow/timing. For each domain, the risk of bias and concerns regarding applicability were rated as “low,” “high,” or “unclear.” Any disagreements were resolved by consensus or through consultation with a third reviewer.

### 2.5 Statistical analysis

Meta-analyses were conducted via a bivariate random-effects model (restricted maximum likelihood (REML)) to pool sensitivity, specificity, PLR, NLR, and DOR, with corresponding 95% confidence intervals (CIs) and 95% prediction intervals (PIs), reflecting expected performance in future settings. A summary ROC (SROC) curve was generated, and the AUC and its 95% CI were estimated directly from the bivariate model. Diagnostic accuracy was classified as “fair” (0.70–0.80), “good” (0.80–0.90), or “excellent” (>0.90) (16). Threshold effects were assessed via Spearman’s correlation between logit(sensitivity) and logit(1–specificity) (*ρ* > 0.60 and *p* < 0.05 indicating a potential threshold effect) and visual inspection of the SROC curve for a “shoulder–arm” pattern. Nontreshold heterogeneity was evaluated via Cochran’s Q, Higgins’ I^2^, and τ^2^ statistics, with low heterogeneity defined as Q *p* > 0.10, I^2^ < 50%, and τ^2^ ≈ 0.

The sensitivity analyses included leave-one-out procedures and alternative data substitution when multiple eligible 2 × 2 datasets were available within a study. Several prespecified effect modifiers—patient positioning, measurement timing, assessor qualifications, and variability in diagnostic thresholds—were considered for subgroup analyses or meta-regression to explore potential sources of heterogeneity. Consistent with the recommended practice for diagnostic test accuracy meta-analyses, these analyses were planned *a priori* but were undertaken only when ≥10 studies were available per predictor. Publication bias was assessed via Deeks’ funnel plot regression (*p* > 0.05 indicating no small-study effects). Clinical applicability was evaluated via Fagan nomograms, which apply the pooled PLR/NLR to the summary prevalence of FI. Risk-of-bias assessments were performed using Review Manager (v5.4), whereas all other statistical analyses were conducted in R (v4.5.0), with a two-sided *α* = 0.05.

## 3 Results

### 3.1 Study selection and characteristics

**Figure 1:**
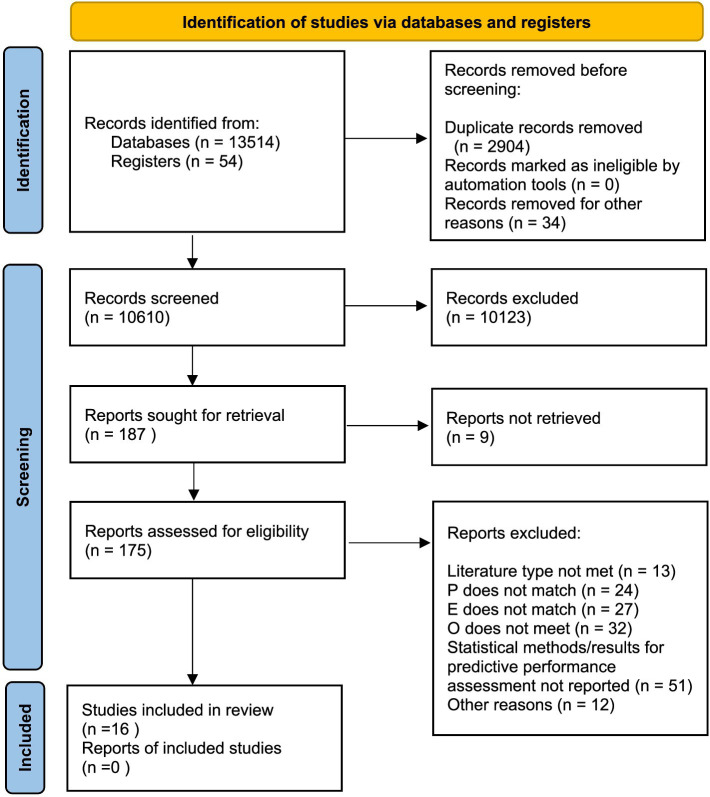
Flow chart of study selection.

Sixteen of the 13,568 records identified through database and supplementary searches met the inclusion criteria (Figure 1). These studies, published between 2019 and 2024, included 12 prospective cohort studies and 4 retrospective designs; 3 were multicenter studies, and 13 were single-center studies. Ten studies were conducted in China, 2 in India, 2 in Turkey, and 1 each in France and Mexico.

The pooled cohort included 1,261 critically ill adults (mean age 37.42–77.03 years; 41.32% female). The follow-up durations varied: 9 studies monitored patients for 1–7 days after EN initiation, 2 until the completion of EN, 3 for 5–7 days post-ICU admission, 1 until hospital discharge, and 1 with an unspecified follow-up duration. EN was delivered via gastric tubes (*n* = 10), gastric or postpyloric tubes (*n* = 2), or unspecified routes (*n* = 4). Funding was reported in 10 studies, not reported in 5, and undisclosed in 1. The detailed study-level and participant characteristics are summarized in Table 1.

**Table 1:** Characteristics of the included studies and participants.

First Author year	Country	Study design	Centers (*n*)/Sample size (*N*)	Setting	GI-US parameters reported	Outcome(s)	FI-directed interventions	Age, years (mean ± SD)	Sex (M/F), *n*
Ankalagi B 2022 (9)	India	Prospective	1/130	Mixed ICUs	②	FI	Not implemented	37.42 ± 16.46	78/52
Chen B 2024 (10)	China	Retrospective	1/108	ICU	⑩	FI; Intestinal mucosal barrier function (DAO, LPS)	Not reported	73.16 ± 13.00	58/50
Chen C 2020 (18)	China	Retrospective	1/42	EICU	①	FI	Not reported	53.00 ± 13.00	31/11
El Khoury D 2023 (7)	France	Prospective	2/44	ICUs	①	FI (gastric intolerance)	Not reported	62.10 ± 10.90	32/12
Fu H 2024 (28)	China	Prospective	1/82	Central ICU, Neurology ICU	⑪	FI	Not reported	55.50 ± 16.25	52/30
Gao T 2019 (11)	China	Prospective	1/116	Surgical ICU	③, ④, ⑤, ⑥, ⑦, ⑫, ⑬	FI	Implemented	53.40 ± 20.60	62/54
Lai J 2022 (19)	China	Retrospective	1/105	ICU	①, ③, ④	FI (EN failure)	Implemented	71.38 ± 8.18	65/40
Li T 2024 (20)	China	Prospective	1/30	General ICU	①, ②	FI	Implemented	77.03 ± 13.55	13/17
Onuk S 2023 (21)	Turkey	Prospective	1/39	ICU Center	①	FI (gastrointestinal dysfunction)	Not reported	60.00 ± 22.96	20/19
Pérez-Calatayud AA 2022 (24)	Mexico	Prospective	2/61	Mixed ICUs	①, ②	FI	Not reported	49.73 ± 10.96	25/36
Sharma R 2023 (25)	India	Prospective	1/57	ICU	②	FI	Implemented	50.50 ± 14.10	32/25
Taskin G 2021 (22)	Turkey	Prospective	1/56	Tertiary ICU	①, ⑧	FI (aspirated GRV ≥ 250 mL)	Not reported	75.14 ± 15.59	27/29
Wang L 2022 (27)	China	Prospective	1/38	Central ICU	①, ⑨	FI	Implemented	61.00 ± 14.40	24/14
Xiang M 2024 (26)	China	Retrospective	1/112	ICU	②	FI	Not reported	73.02 ± 5.83	62/50
Yu G 2023 (8)	China	Prospective	1/91	ICU	①, ⑫, ⑭	FI	Implemented	65.70 ± 15.00	60/31
Zou T 2019 (23)	China	Prospective	5/150	Tertiary ICU	①	FI	Not reported	58.63 ± 16.45	99/51

EN, enteral nutrition; FI, feeding intolerance; DAO, diamine oxidase; LPS, lipopolysaccharide; ICU, intensive care unit; EICU, emergency ICU.

Ultrasound indicators: ① Gastric antrum cross-sectional area (GCSA); ② gastric residual volume (GRV); ③ intestinal diameter; ④ intestinal peristalsis; ⑤ intestinal wall thickness; ⑥ alterations in intestinal folds; ⑦ intestinal wall stratification; ⑧ anteroposterior (dAP) and craniocaudal (dCC) diameters of the gastric antrum; ⑨ gastric antrum wall echodensity; ⑩ blood flow velocity in the superior mesenteric artery (SMA), including peak systolic velocity (PSV), end-diastolic velocity (EDV), and pulsatility index (PI); ⑪ antral motility index (MI); ⑫ acute gastrointestinal injury ultrasonography (AGIUS) score; ⑬ gastrointestinal and urinary tract sonography (GUTS) protocol score; and ⑭ transabdominal gastrointestinal ultrasonography (TGIU) parameters.

### 3.2 Methodological quality assessment

**Figure 2:**
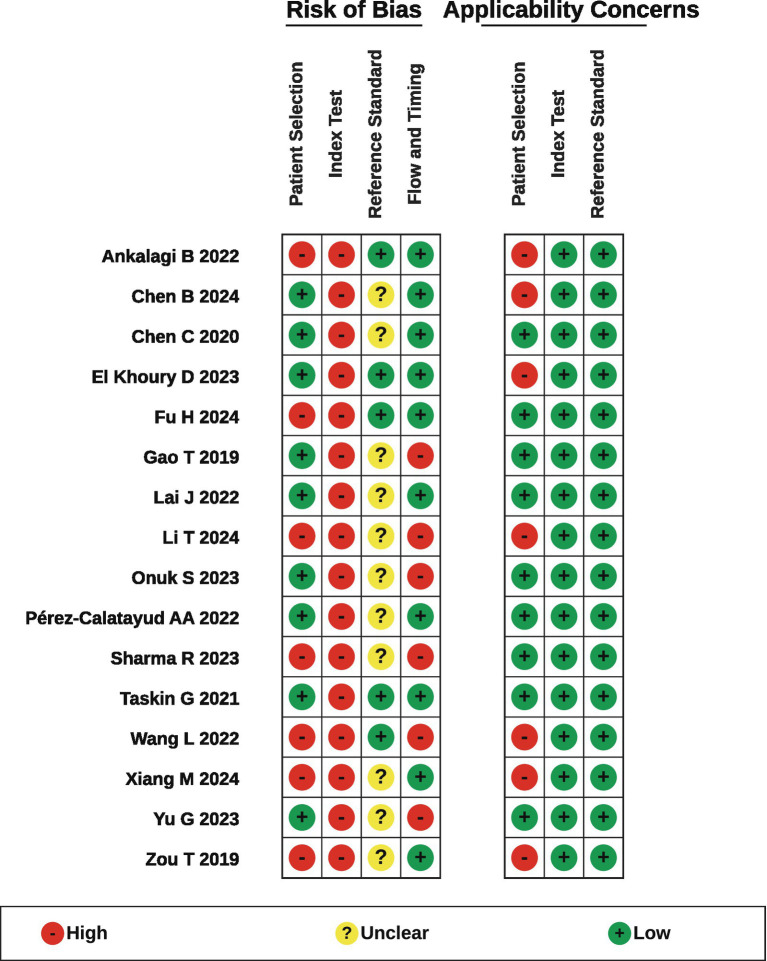
QUADAS-2 bias and applicability assessment.

The overall methodological quality varied (Figure 2). The interrater agreement for the QUADAS-2 judgments was substantial (*κ* = 0.65; 95% CI: 0.51–0.77) (17).

#### 3.2.1 Patient selection

Seven studies (43.75%) were at high risk of bias due to nonconsecutive/nonrandom recruitment or unclear reporting. Several common ICU contexts related to FI (e.g., small bowel feeding, early postoperative status, and abdominal distension) were excluded, limiting generalizability.

#### 3.2.2 Index test

All studies (100%) were at high risk of bias due to the use of *post hoc*, ROC-based data-driven thresholds rather than prespecified cutoff values. In addition, several studies did not report whether GI-US operators or image interpreters were blinded to the FI adjudication process.

#### 3.2.3 Reference standards

Eleven studies (68.75%) were at unclear risk because blinding of FI adjudicators to GI-US procedures or results was not reported; the remaining studies explicitly conducted FI determination independently and were therefore rated low risk.

#### 3.2.4 Flow and timing

Six studies (37.50%) were high risk due to incomplete follow-up/attrition or exclusion of poor-quality scans without adjustment.

#### 3.2.5 Applicability concerns

Seven studies (43.75%) focused on patient selection (restricted populations). No applicability concerns were identified for the index test or reference standard.

##### 3.2.5.1 Patient selection

Seven studies (43.75%) raised applicability concerns regarding patient selection. These concerns arose primarily from the exclusion of participants with specific clinical conditions—such as small bowel feeding, early postoperative status, or abdominal distension—which limited population representativeness and reduced alignment with common ICU EN scenarios.

##### 3.2.5.2 Index test

No applicability concerns were identified for the index test (GI-US). The operational procedures and interpretative processes for GI-US were consistent with its anticipated use in routine clinical practice.

##### 3.2.5.3 Reference standards

No applicability concerns were identified for the reference standard (FI). The diagnostic criteria employed were those routinely used in contemporary ICU settings.

### 3.3 Categorized GI-US parameters

Across the 16 studies, 10 single GI-US parameters were identified, which can be grouped by anatomical/physiological origin: (1) gastric: GCSA, GRV, gastric antral wall echodensity, and antral motility index (AMI); (2) intestinal: intestinal diameter, intestinal peristalsis, intestinal wall thickness, alterations in intestinal folds, and intestinal wall stratification; and (3) SMA: SMA flow velocities. The acquisition protocols and study-level diagnostic performance for all the predictors are summarized in Supplementary Table 3.

### 3.4 Diagnostic performance of single GI-US parameters

#### 3.4.1 Diagnostic accuracy of GCSA [primary analysis]

The GCSA was the only GI-US predictor meeting the prespecified threshold for quantitative synthesis and thus was selected for the primary analysis. Nine studies (*n* = 619) evaluated the GCSA; eight studies (7, 8, 18–23) (*n* = 558) directly reported its diagnostic performance and were considered for pooling. One study (24) (*n* = 61) assessed the change in the GCSA (ΔGCSA) at different time points but provided limited evidence (see Supplementary Table 4). Among the eight studies that directly measured the GCSA, all but Taskin (22) defined FI via a composite reference (elevated GRV, clinical symptoms, and/or inadequate EN). Taskin et al. (22) used GRV ≥ 250 mL as a single criterion and was thus excluded from the meta-analysis to reduce heterogeneity and improve interpretability.

##### 3.4.1.1 Pooled performance

**Figure 3:**
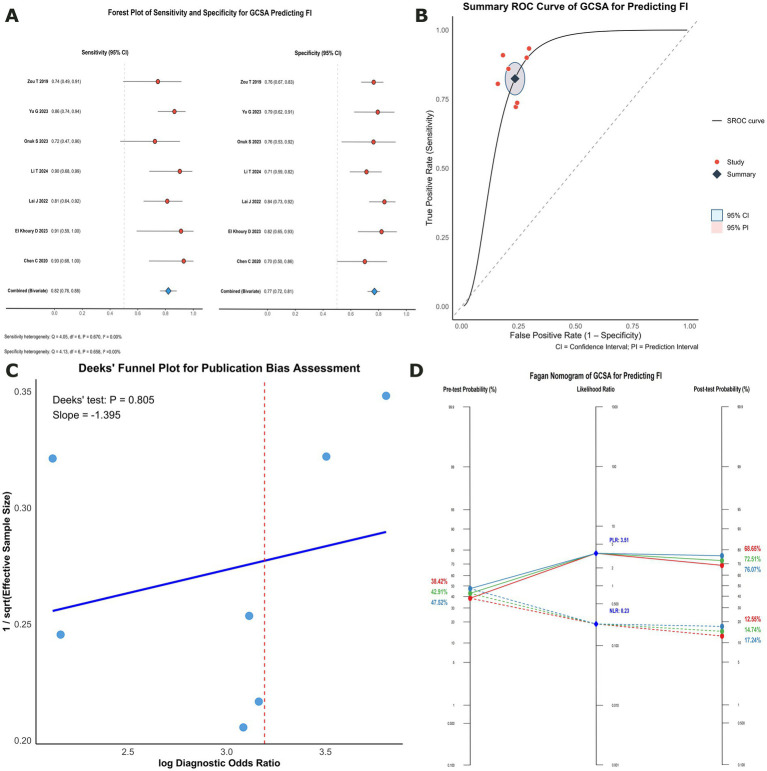
Diagnostic performance of the GCSA for predicting FI. **(A)** Forest plot of sensitivity and specificity; **(B)** Summary receiver operating characteristic (SROC) curve; **(C)** Deeks’ funnel plot assessing small-study effects; **(D)** Fagan nomogram showing post-test probabilities. Point estimates were recalculated from reconstructed 2 × 2 tables.

Seven studies (7, 8, 18–21, 23) (*n* = 502) were pooled via a bivariate random-effects model based on reconstructed 2 × 2 tables. Chen (18) reported three patient positions: supine, semireclining, and right lateral decubitus (RLD). As the 2 × 2 tables for the semireclining and RLD positions were identical and aligned most closely with those used in other studies, these datasets were incorporated into the primary analysis, and the supine-position data were retained for sensitivity analyses. Overall, the GCSA demonstrated good diagnostic accuracy, with an AUC of 0.86 (95% CI 0.76–0.89; 95% PI 0.76–0.89). The pooled sensitivity was 0.82 (95% CI 0.76–0.88; 95% PI 0.76–0.88), the specificity was 0.77 (95% CI 0.72–0.81; 95% PI 0.72–0.81), the DOR was 15.29 (95% CI 9.56–24.46), the PLR was 3.51 (95% CI 2.90–4.26), and the NLR was 0.23 (95% CI 0.17–0.32). The forest plots and SROC curves are shown in Figures 3A,B.

##### 3.4.1.2 Heterogeneity

Spearman’s correlation coefficient of 0.39 (*p* = 0.396) indicated no significant threshold effect, which was consistent with the SROC shape. Nontreshold heterogeneity was low: sensitivity (Q = 4.05, *p* = 0.670; I^2^ = 0%), specificity (Q = 4.13, *p* = 0.658; I^2^ = 0%), and log DOR (Q = 3.38, *p* = 0.760; I^2^ = 0%). Similarly, the variance components (τ^2^_sensitivity = 0.00; τ^2^_(1 − specificity) = 0.00) suggested minimal between-study heterogeneity. Given that fewer than ten studies were available for this predictor and that between-study heterogeneity was minimal, prespecified subgroup analyses and meta-regression were not performed.

##### 3.4.1.3 Sensitivity analyses

Leave-one-out analyses revealed stable estimates: sensitivity, 0.81–0.84; specificity, 0.75–0.78; AUC, 0.77–0.88; DOR, 13.48–17.60; PLR, 3.34–3.68; and NLR, 0.21–0.25 (Supplementary Table 5; Supplementary Figures 1, 2). Replacing Chen’s (18) semirecumbent/right-lateral data with supine data yielded similar results (AUC 0.86 [95% CI 0.77–0.89], sensitivity 0.80 [95% CI 0.73–0.86], specificity 0.78 [95% CI 0.73–0.83], DOR 14.54 [95% CI 9.11–23.20], PLR 3.68 [95% CI 3.00–4.50], NLR 0.25 [95% CI 0.19–0.34]).

##### 3.4.1.4 Publication bias

Deeks’ funnel plot regression indicated no small-study effects (slope −1.40; *p* = 0.805) (Figure 3C).

##### 3.4.1.5 Clinical applicability

Assuming a pretest probability of FI of 42.91% (95% CI 38.42–47.52%) (5), the pooled PLR/NLR translated to a posttest probability of 72.51% (95% CI 69.73–76.10%) after a positive GCSA and 14.74% (95% CI 11.99–17.84%) after a negative GCSA. The results of the scenario analyses for extreme pretest probabilities are shown in Figure 3D.

#### 3.4.2 Diagnostic accuracy of other single GI–US parameters [secondary analysis]

Apart from the GCSA, the remaining single parameters did not meet the prespecified threshold for pooling due to limited study numbers or inconsistent reporting. A structured descriptive synthesis is therefore presented by anatomical origin. According to the predefined AUC categories, the overall diagnostic performance ranged from fair to excellent. However, although several predictors demonstrated good to excellent AUC values, the overall evidence remains limited by small sample sizes, inconsistent protocols, heterogeneous thresholds and measurement time points, and a lack of external validation.

##### 3.4.2.1 Gastric parameters

Three studies (9, 25, 26) (*n* = 299) evaluated the GRV and reported AUC values of 0.77–0.99, indicating good to excellent diagnostic accuracy. Notably, at 4 h after EN initiation, Ankalagi (9) reported excellent performance (AUC 0.99, sensitivity 1.00, specificity 0.99). One study (27) (*n* = 38) assessed gastric antral wall echodensity and yielded fair performance (AUC 0.75–0.76). Another study (28) (*n* = 82) evaluated AMI and reported good performance (AUC 0.86).

##### 3.4.2.2 Intestinal parameters

Two studies (11, 19) (*n* = 221) investigated intestinal diameter and reported AUC values ranging from 0.60 to 0.92 (fair to excellent). Intestinal peristalsis, assessed in the same two studies, showed good diagnostic accuracy (AUC 0.78–0.85). One study (11) (*n* = 116) examined intestinal wall characteristics—including wall thickness, folds, and stratification—and yielded fair diagnostic performance (AUC 0.71–0.77).

##### 3.4.2.3 SMA parameters

One study (10) (*n* = 108) assessed SMA velocities. The AUC values were 0.74–0.77 for peak systolic velocity (PSV) and 0.71–0.80 for end-diastolic velocity (EDV), indicating good diagnostic accuracy.

### 3.5 Diagnostic performance of the composite ultrasound score and multivariable models [exploratory analysis]

In addition to single predictors, two studies developed three composite score or multivariable models integrating gastric and/or intestinal parameters. Owing to the limited number of studies, these models were not meta-analyzed and were summarized descriptively.

#### 3.5.1 Acute gastrointestinal injury ultrasonography score

One study (8) (*n* = 91) proposed the AGIUS, which integrates the intestinal diameter, wall thickness, and peristalsis frequency, achieving an AUC of 0.83, indicating good diagnostic accuracy.

#### 3.5.2 Transabdominal gastrointestinal ultrasonography model

The same study combined the AGIUS with the GCSA to develop the TGIU model, which improved the AUC to 0.88 (good).

#### 3.5.3 PRE series models

Another study (19) (*n* = 105) introduced the PRE series. The optimal combination (GCSA + intestinal diameter + intestinal peristalsis) achieved an AUC of 0.95 (excellent). Other combinations yielded AUCs of 0.95 (GCSA + diameter), 0.93 (GCSA + peristalsis), and 0.91 (diameter + peristalsis).

Taken together, single GI-US parameters demonstrated fair to good diagnostic accuracy, with the GRV showing the highest reported performance at specific time points and the GCSA providing the most robust pooled evidence. The composite predictors consistently outperformed the single-parameter measures (*p* < 0.05) (Table 2).

**Table 2:** Diagnostic performance of additional GI-US predictors for FI.

GI-US predictors	Study	Specific predictors	Sample size for prediction	AUC	Sensitivity	Specificity	PLR	NLR	Cutoff	*p* value
GRV	Ankalagi B, 2022 (9)	GRV1 (At the end of 1 h of EN initiation)	130	0.94	0.92	0.88	7.67	0.09	49.9 mL	0.021
		GRV2 (At the end of 2 h of EN initiation)		0.92	0.85	0.84	5.31	0.18	86.8 mL	0.042
		GRV3 (At the end of 3 h of EN initiation)		0.96	0.92	0.91	10.22	0.09	148.0 mL	<0.012
		GRV4 (At the end of 4 h of EN initiation)		0.99	1.00	0.99	100.00	0.00	216.7 mL	<0.014
	Sharma R, 2023 (25)	GRV estimation by the USG method	57	0.82	0.97	0.98	48.50	0.03	NR	<0.05
	Xiang M, 2024 (26)	GRV	112	0.77	0.74	0.72	2.64	0.36	NR	<0.05
Intestinal diameter	Gao T, 2019 (11)	Daily within 1 week after ICU admission (8:30–10:00 a.m.)	116	0.60	NR	NR	NR	NR	NR	<0.05
	Lai J, 2022 (19)	EN initiation	105	0.92	0.75	0.91	8.33	0.27	≤2.9 cm	<0.05
Intestinal peristalsis	Gao T, 2019 (11)	Daily within 1 week after ICU admission (8:30–10:00 a.m.)	116	0.78	NR	NR	NR	NR	NR	<0.05
	Lai J, 2022 (19)	EN initiation	105	0.85	0.86	0.71	2.97	0.20	>3	<0.05
Thickness of the intestinal wall	Gao T, 2019 (11)	Daily within 1 week after ICU admission (8:30–10:00 a.m.)	116	0.71	NR	NR	NR	NR	NR	<0.05
Alterations in intestinal folds	Gao T, 2019 (11)	Daily within 1 week after ICU admission (8:30–10:00 a.m.)	116	0.76	NR	NR	NR	NR	NR	<0.05
Intestinal wall stratification	Gao T, 2019 (11)	Daily within 1 week after ICU admission (8:30–10:00 a.m.)	116	0.77	NR	NR	NR	NR	NR	<0.05
Gastric antral wall echodensity Wall-ED50	Wang L, 2022 (27)	Every morning before feeding for the first 7 days after ICU	38	0.76	0.69	0.8	3.45	0.39	63	0.006
Echodensity of the gastric antrum Wall-ED85	Wang L, 2022 (27)	Every morning before feeding for the first 7 days after ICU	38	0.75	0.69	0.75	2.76	0.41	77.5	0.006
Echodensity of the gastric antrum Wall-EDmean	Wang L, 2022 (27)	Every morning before feeding for the first 7 days after ICU	38	0.76	0.69	0.88	5.75	0.35	65.9	0.004
SMA-PSV	Chen B, 2024 (10)	Day1 after EN initiation	108	0.75	0.86	0.53	1.83	0.26	NR	<0.05
		Day3 after EN initiation	108	0.77	0.49	0.92	6.13	0.55	NR	<0.05
		Day7 after EN initiation	108	0.74	0.73	0.70	2.43	0.39	NR	<0.05
SMA-EDV	Chen B, 2024 (10)	Day1 after EN initiation	108	0.77	0.96	0.51	1.96	0.08	NR	<0.05
		Day3 after EN initiation	108	0.80	0.80	0.80	4.00	0.25	NR	<0.05
		Day7 after EN initiation	108	0.71	0.69	0.68	2.16	0.46	NR	<0.05
AMI	Fu H, 2024 (28)	Day3 after EN initiation	82	0.86	0.77	0.78	3.50	0.29	0.556	<0.05
AGIUS	Yu G, 2023 (8)	Day1, 3, 5, 7 after EN initiation	91	0.83	0.88	0.82	4.89	0.15	3.5 points	<0.001
TGIU	Yu G, 2023 (8)	Day1, 3, 5, 7 after EN initiation	91	0.88	NR	NR	NR	NR	NR	<0.001
PRE [GCSA+Diam+Peri]	Lai J, 2022 (19)	EN initiation	105	0.95	0.89	0.89	8.09	0.12	>0.68	<0.05
PRE [GCSA+Diam]	Lai J, 2022 (19)	EN initiation	105	0.95	0.86	0.88	7.17	0.16	>0.58	<0.05
PRE [GCSA+Peri]	Lai J, 2022 (19)	EN initiation	105	0.93	0.85	0.87	6.54	0.17	>0.55	<0.05
PRE [Diam+Peri]	Lai J, 2022 (19)	EN initiation	105	0.91	0.88	0.77	3.83	0.16	>0.65	<0.05

FI, feeding intolerance; EN, enteral nutrition; GRV, gastric residual volume; AMI, antral motility index; SMA, superior mesenteric artery; PSV, peak systolic velocity; EDV, end-diastolic velocity; AGIUS, acute gastrointestinal injury ultrasonography score; TGIU, transabdominal gastrointestinal ultrasonography; GCSA, gastric antrum cross-sectional area; Diam, intestinal diameter; Peri, intestinal peristalsis; NR, not reported. Data are directly extracted from the original studies without reconstruction of 2 × 2 contingency tables due to incomplete raw data. The PLR and NLR are omitted where not reported.

## 4 Discussion

### 4.1 Methodological quality of the included studies

The QUADAS-2 assessment revealed that the overall quality of the available evidence was moderate, with several significant methodological limitations. (1) Participant enrollment and representativeness: In total, 43.75% of the studies did not employ consecutive or random enrollment and excluded patients in common ICU scenarios, such as small bowel feeding and postoperative abdominal distension. This could be attributed to the complexity of the ICU case mix, feasibility constraints, and ethical considerations. Given that this field remains exploratory and that GI-US protocols have yet to be standardized (29), some studies further excluded patients with limited sonographic windows to maintain image quality and consistency; however, this inevitably reduced the sample representativeness and weakened the external generalizability. (2) Blinding and threshold definitions: Most studies did not clearly report blinding methods, and in more than half of them, it was unclear whether GI-US operators and FI adjudicators were mutually blinded, reflecting shortcomings in the design of the blinding procedures and the transparency of reporting. In the absence of unified GI–US threshold standards, many studies have adopted ROC curve-based, data-driven cutoff values to optimize model performance. However, this *post hoc* determination may have led to overestimation of diagnostic accuracy and compromised external reproducibility (30). (3) Loss to follow-up and selective reporting: Approximately 37.50% of the studies did not analyze high loss-to-follow-up rates (≥10%) or directly excluded cases with poor-quality images. This could be due to significant fluctuations in ICU patients’ clinical status, limited positioning options, and abdominal distension, all of which can compromise image quality (29). Some studies, in an effort to preserve data consistency and model stability, preferentially excluded such cases without employing missing-data imputation or conducting bias analyses. Notably, patients with poor-quality images or those lost to follow-up are often more severely ill and at higher risk for FI. Excluding these patients could systematically underestimate the true incidence of FI and overestimate model performance, thus increasing the risk of selective reporting bias.

Taken together, the limitations in participant enrollment, blinding, and data completeness underscore the need for caution when extrapolating these findings to different populations and clinical contexts. Nonetheless, it is important to note that, aside from concerns regarding population representativeness, the operational procedures for GI-US and the clinical criteria used to diagnose FI were highly consistent with routine ICU practices, indicating a relatively low risk of applicability concerns. Therefore, when measurement and diagnostic pathways align with standard clinical care, the existing evidence remains broadly applicable to most clinical settings, despite variations in patient populations.

### 4.2 Systematic mapping of GI-US predictors and their comparative performance

Across the existing studies, approximately ten GI-US predictors related to the ENFI have been identified and categorized into gastric, intestinal, and SMA parameters. Gastric predictors are supported by the most substantial evidence base, whereas intestinal and SMA-related parameters, despite having strong physiological and mechanistic foundations, remain in the exploratory stage with relatively limited supporting data.

#### 4.2.1 Gastric predictors

All identified gastric predictors are derived from measurements of the gastric antrum. The GCSA is the most widely applied and methodologically established quantitative parameter, reflecting gastric emptying efficiency and the degree of chyme retention, and serves as a key indicator of gastric motility. As delayed gastric emptying is an early sign of FI, increases in the GCSA may indicate motility impairment before the onset of symptoms, providing strong physiological justification and predictive potential (7). Other gastric measures, such as the GRV, AMI, and gastric wall echogenicity, show varying degrees of association (28, 29, 31), but the evidence supporting these parameters is still limited. Overall, the GCSA offers the greatest advantages in terms of stability and reproducibility, representing a central focus for future research and clinical translation.

#### 4.2.2 Intestinal predictors

The intestine, as the primary site of nutrient absorption in EN, plays a crucial role in the development of FI through changes in structure and motility (32). Ultrasonographic parameters such as bowel diameter, peristalsis, wall thickness, mucosal folds, and layer stratification reflect both motility and barrier function of the intestine, with alterations often occurring before clinical symptoms appear, providing predictive value (33–36). However, current studies are limited by small sample sizes and inconsistent parameter acquisition protocols, leading to significant variability in predictive performance. The AUCs for bowel diameter and peristalsis vary widely (0.60–0.92 and 0.78–0.85, respectively), indicating potential but insufficient stability. The evidence for wall thickness and structural parameters is more limited. These limitations likely stem from factors such as technical complexity, interference from bowel gas, and the lack of standardized measurement protocols (37–39). Future research should focus on standardizing measurement techniques and parameter definitions while integrating dynamic monitoring and multitime-point validation to improve the stability and reproducibility of intestinal predictors.

#### 4.2.3 SMA predictors

SMA parameters reflect gastrointestinal function from a perfusion perspective, with variations in blood flow velocity offering indirect insights into mesenteric perfusion and absorptive capacity (40, 41). Existing studies have demonstrated that the AUCs for the PSV and EDV generally range from 0.71 to 0.80 (fair–good), indicating moderate discriminatory power. However, the current evidence is insufficient to draw robust conclusions. As potential hemodynamic biomarkers, SMA parameters may, in the future, be used in conjunction with gastric motility and intestinal structural measures to develop composite models, thus improving overall predictive performance and clinical interpretability.

### 4.3 Diagnostic performance and clinical applicability of the GCSA

Current evidence shows that the GCSA has excellent and stable diagnostic performance in predicting ENFI, making it the most representative GI-US parameter to date. The results from the bivariate random-effects model in this study showed an AUC of 0.86 (95% CI: 0.76–0.89), with a sensitivity and specificity of 0.82 and 0.77, respectively, underscoring the high discriminative power of the GCSA and its ability to identify high-risk ENFI patients effectively at an early stage. Clinically, the GCSA can provide a quantitative risk assessment for ENFI. A positive result (posttest probability of approximately 72.51%) indicates a higher risk of FI and should prompt clinical attention with appropriate adjustments to the EN regimen. In contrast, a negative result (approximately 14.74%) suggests a lower short-term risk, supporting continued nutritional advancement and dynamic reassessment (42). The risk stratification model based on posterior probability helps facilitate personalized EN management, reducing unnecessary feeding interruptions and malnutrition. However, the GCSA should be regarded as an adjunctive decision-making tool rather than an independent diagnostic standard and must be interpreted alongside the patient’s overall condition and institutional guidelines.

From a methodological standpoint, this study further confirms the robustness and reliability of the diagnostic performance of the GCSA. This comprehensive analysis not only validates the clinical application potential of the GCSA but also strengthens the methodological robustness of the evidence. In contrast to previous studies, which were primarily single-center studies and reported only a single performance metric, this study systematically assessed multiple diagnostic parameters, including sensitivity, specificity, AUC, DOR, and likelihood ratios, using a bivariate model and conducted heterogeneity and sensitivity analyses, providing more clinically interpretable and reliable evidence. Although the results show stable performance for the GCSA, discrepancies across studies regarding measurement position, threshold values, and time windows limit the establishment of unified operational thresholds, affecting its direct applicability across different centers and populations. Thus, the findings of this study should be considered preliminary validations of the robustness of the GCSA rather than conclusive evidence for its universal applicability across all clinical contexts. Notably, the threshold differences across the included studies had minimal impact on overall performance (Spearman’s *ρ* = 0.39, *p* = 0.396), indicating that the GCSA has high consistency and generalizability.

In addition to diagnostic performance, the clinical feasibility of the GCSA has also been demonstrated in several studies. Its key advantages include the following: (1) ease of acquisition: ICU nurses can master the standardized measurement protocol after approximately 4 hours of structured training (43), with good interobserver agreement (ICC = 0.87) (44), and a single measurement takes approximately 5 min (45); (2) high standardization: probe positioning, measurement planes, and anatomical landmarks are standardized in the ASPEN and ESPEN guidelines (2, 3), ensuring high repeatability and cross-center comparability; and (3) strong accessibility: bedside ultrasound equipment is widely available, noninvasive, and allows for dynamic monitoring of critically ill patients. In summary, the GCSA combines robust diagnostic performance with excellent operability, making it the most promising single GI-US parameter for clinical translation. Future research should focus on threshold standardization, dynamic monitoring strategies, and cross-population validation to enhance comparability across settings and operational feasibility, ultimately enabling its transition from a research tool to a clinical decision-making instrument.

### 4.4 Potential advantages of the composite ultrasound score and multivariable models

While the number of relevant studies remains limited, precluding a quantitative meta-analysis, several exploratory studies (2, 3) have provided preliminary validation of the value of composite ultrasound scoring and multivariable models (e.g., AGIUS, TGIU, PRE) in predicting ENFI. The AUCs typically fall within the “good to excellent” range, with other diagnostic performance metrics also demonstrating superior predictive ability compared with individual parameters. From both mechanistic and methodological perspectives, the potential advantages of composite models can be highlighted in two key areas. First, the integration of multiple parameters may enhance the model’s discriminative power and robustness. By combining various ultrasound features (e.g., GCSA, bowel diameter, peristalsis), such models have the theoretical capacity to capture pathophysiological information across multiple levels, potentially overcoming the limitations of individual parameters and thereby facilitating more comprehensive risk identification. Second, multidimensional integration is expected to improve the model’s physiological interpretability. Incorporating parameters from various anatomical sites and physiological dimensions (e.g., motility, structural barriers, blood flow perfusion) may help systematically characterize the pathophysiological processes underlying ENFI.

This integrative approach aligns with the current trend in predictive modeling toward “multimodal, multidimensional fusion” (46), which aims to reduce the impact of individual variability, technical errors, and local lesions on model performance, thereby enhancing both external validity and clinical applicability. Nevertheless, these advantages remain largely theoretical, as empirical evidence is still limited. Most existing models are underdeveloped in terms of their coverage of physiological dimensions, variable selection, and weighting. For example, SMA parameters reflecting mesenteric perfusion have yet to be systematically integrated, and feature selection frequently relies on expert judgment rather than a standardized or data-driven process, limiting the model’s interpretability and cross-center applicability. Overall, while composite models show considerable theoretical and methodological promise, the current evidence base remains exploratory. Further empirical validation is needed, particularly in the areas of feature standardization, transparency in variable selection, and external validation.

### 4.5 Limitations and future directions

While this study provides a comprehensive assessment of the application of GI-US in predicting ENFI, several limitations remain because of the systematic integration of existing evidence. With the exception of the GCSA, the number of studies and sample sizes for most indicators were insufficient to support quantitative meta-analysis. Additionally, many of the included studies faced challenges such as nonconsecutive participant enrollment, insufficient blinding, and inconsistent threshold definitions, which limited the robustness of the evidence and its external generalizability. Even for the GCSA, there is no consensus on optimal cutoff values, measurement positions, or time windows, complicating the establishment of directly applicable clinical standards. Furthermore, most of the existing studies were single-center, small-sample designs, with the majority (10/16) conducted in China, raising concerns about regional differences and heterogeneity in operational protocols, which could impact the generalizability of the results. GI–US measurements also remain operator dependent; although preliminary evidence suggests good consistency, further validation of reproducibility across centers and varying levels of experience is still needed.

Overall, while the existing evidence is still in the exploratory phase, the consistent direction of the findings provides relatively reliable support for the potential of GI-US in ENFI risk prediction. Future research should focus on two key areas: first, prospective, multicenter, large-sample studies to optimize threshold settings, standardize protocols, and improve blinding methods to increase reproducibility and external validity; second, the development of modeling strategies that combine physiological mechanisms with data-driven approaches, integrating multidimensional ultrasound features (such as gastric motility, intestinal structure, and blood flow perfusion) to build validated composite predictive models while leveraging artificial intelligence to optimize feature selection and weight distribution. Through a collaborative effort in standardization, validation, and automation, GI-US has the potential to transition from a research tool to a clinical decision support system, providing actionable, evidence-based guidance for the early identification and precise intervention of ENFI.

## 5 Conclusion

This systematic review and meta-analysis provides a comprehensive evaluation of the application of GI-US in predicting ENFI in critically ill adults, systematically integrating diagnostic performance evidence for various indicators. The findings reveal that the available GI-US predictors can be categorized into three anatomical domains: gastric (predominantly GCSA), intestinal, and SMA parameters. Among these, the GCSA stands out for its robust diagnostic performance and strong clinical feasibility, making it the most well-supported and clinically translatable single indicator. In contrast, while evidence for other single predictors and composite models remains limited, multisite, multiparameter fusion strategies show considerable potential to increase predictive accuracy. These strategies provide a clear direction for developing integrated models that combine gastric motility, intestinal responsiveness, and perfusion status. Future research should focus on large-scale, multicenter prospective cohorts to further validate and optimize relevant indicators, standardize measurement conditions and threshold settings, and integrate artificial intelligence-driven real-time monitoring and decision support systems to enable precise, standardized, and proactive management of ENFI.

## Data Availability

The original contributions presented in the study are included in the article/Supplementary material, further inquiries can be directed to the corresponding author.

## Glossary

AGIUS: Acute Gastrointestinal Injury Ultrasonography
AMI: Antral Motility Index
ASPEN: American Society for Parenteral and Enteral Nutrition
AUC: Area Under the Curve
CI: Confidence Interval
CNKI: China National Knowledge Infrastructure
CT: Computed Tomography
DAO: Diamine Oxidase
DOR: Diagnostic Odds Ratio
EDV: End-Diastolic Velocity
EICU: Emergency Intensive Care Unit
EN: Enteral Nutrition
ENFI: Enteral Feeding Intolerance
ESPEN: European Society for Parenteral and Enteral Nutrition
FI: Feeding Intolerance
GCSA: Gastric Antrum Cross-Sectional Area
GI-US: Gastrointestinal ultrasound
GRV: Gastric Residual Volume
ICU: Intensive Care Unit
LPS: Lipopolysaccharide
NLR: Negative Likelihood Ratio
NR: Not Reported
PI: Prediction Interval
PLR: Positive Likelihood Ratio
PRISMA-DTA: Preferred Reporting Items for Systematic Reviews and Meta-Analyses of Diagnostic Test Accuracy
PSV: Peak Systolic Velocity
QUADAS-2: Quality Assessment of Diagnostic Accuracy Studies-2
REML: Restricted Maximum Likelihood
SMA: Superior Mesenteric Artery
SROC: Summary Receiver Operating Characteristic
TGIU: Transabdominal Gastrointestinal Ultrasonography
