# Supplementary material for: Diagnostic accuracy of gastrointestinal ultrasound in predicting enteral feeding intolerance: a systematic review and meta-analysis
Source: Front Nutr. 2026 Apr 20;13:1767700. doi: 10.3389/fnut.2026.1767700 (PMC13137820; doi:10.3389/fnut.2026.1767700)
Supplement: Supplementary file 1 [file Supplementary_File_1.DOCX]

Supplementary Material

**Diagnostic Accuracy of Gastrointestinal Ultrasound in Predicting Enteral Feeding Intolerance: A Systematic Review and Meta-analysis**

[SUPPLEMENT FILE CONTENT 1](#_Toc5859)

[Table 1. Search strategy 2](#_Toc19090)

[Table 2. Acute gastrointestinal injury ultrasonography score (AGIUS score) 6](#_Toc20341)

[Table 3. Acquisition and evaluation of GI-US predictors 7](#_Toc5099).

[Table 4. Evaluation Results of the GCSA-FI Predicted Performance 9](#_Toc29785)

[Table 5. Sensitivity analysis of pooled diagnostic metrics via the leave-one-out approach 10](#_Toc5136)

[Fig. 1 Forest plot of leave-one-out sensitivity analysis (sensitivity and specificity)​ 1](#_Toc21144)0

[Fig. 2 Forest plot of leave–one-out sensitivity analysis (positive and negative likelihood ratios)​​ 1](#_Toc23525)1

**Table 1.** Search strategy

| Database | Step | Search strategy |
| --- | --- | --- |
| PubMed | #1 | (Ultrasonography[MeSH Major Topic]) OR (Ultrasonics[MeSH Major Topic]) |
|  | #2 | ultraso*[Title/Abstract] OR sonogra*[Title/Abstract] OR echo*graph*[Title/Abstract] |
|  | #3 | #1 OR #2 |
|  | #4 | Enteral Nutrition[MeSH Major Topic] |
|  | #5 | ((enter*[Title/Abstract] OR digestiv*[Title/Abstract] OR oral[Title/Abstract] OR sip[Title/Abstract] OR nose*[Title/Abstract] OR stomach*[Title/Abstract] OR gastr*[Title/Abstract] OR intragastr*[Title/Abstract] OR nasogastr*[Title/Abstract] OR intestin*[Title/Abstract] OR intraintestin*[Title/Abstract] OR nasointestin*[Title/Abstract] OR nasoenter*[Title/Abstract] OR percutaneous[Title/Abstract] OR esophag*[Title/Abstract] OR esophag*[Title/Abstract] OR duoden*[Title/Abstract] OR jejunostom*[Title/Abstract] OR nasojejun*[Title/Abstract]) AND (nutrition*[Title/Abstract] OR feed*[Title/Abstract] OR support*[Title/Abstract] OR tube*[Title/Abstract] OR intubat*[Title/Abstract])) OR (EN[Title/Abstract]) |
|  | #6 | #4 OR #5 |
|  | #7 | ((((((((((Complications[MeSH Subheading]) OR (Gastric Emptying[MeSH Major Topic])) OR (Energy Intake[MeSH Major Topic])) OR (Diarrhea[MeSH Major Topic])) OR (Flatulence[MeSH Major Topic])) OR (Vomiting[MeSH Major Topic])) OR (Constipation[MeSH Major Topic])) OR (Gastroesophageal Reflux[MeSH Major Topic])) OR (Respiratory Aspiration[MeSH Major Topic])) OR (Gastroparesis[MeSH Major Topic])) OR (Gastrointestinal Hemorrhage[MeSH Major Topic]) |
|  | #8 | (((((((nutrition*[Title/Abstract] OR feed*[Title/Abstract] OR food[Title/Abstract] OR enter*[Title/Abstract] OR tube*[Title/Abstract]) AND (intolera*[Title/Abstract] OR toler*[Title/Abstract] OR fail*[Title/Abstract])) OR ((reflux[Title/Abstract] OR regurgitation[Title/Abstract]) AND (cardio*esophageal[Title/Abstract] OR esophag*[Title/Abstract] OR esophag*[Title/Abstract] OR gastr*[Title/Abstract]))) OR (aspirat*[Title/Abstract] AND ("foreign bod*"[Title/Abstract] OR respiratory[Title/Abstract] OR accidental[Title/Abstract]))) OR ((atoni*[Title/Abstract] OR paresis[Title/Abstract] OR paraly*[Title/Abstract] OR stas*s[Title/Abstract] OR empty*[Title/Abstract] OR residu*[Title/Abstract]) AND (gastric*[Title/Abstract] OR stomach[Title/Abstract]))) OR ((gastro*[Title/Abstract] OR GI[Title/Abstract]) AND (hemorrhage*[Title/Abstract] OR hemorrhage*[Title/Abstract] OR bleeding[Title/Abstract] OR "blood loss"[Title/Abstract]))) OR ((energy[Title/Abstract] OR calor*[Title/Abstract] OR enteral*[Title/Abstract]) AND (intake*[Title/Abstract] OR goal*[Title/Abstract]))) OR (complication*[Title/Abstract] OR sequel*[Title/Abstract] OR enterorrhea[Title/Abstract] OR diarrh*[Title/Abstract] OR flatu*[Title/Abstract] OR vomit*[Title/Abstract] OR emesi*[Title/Abstract] OR constipation*[Title/Abstract] OR "colonic Inertia"[Title/Abstract] OR dyschezia[Title/Abstract] OR obstipation[Title/Abstract] OR GERD[Title/Abstract] OR GORD[Title/Abstract] OR "delayed gastric emptying"[Title/Abstract] OR gastrop*[Title/Abstract] OR hematochezia*[Title/Abstract] OR GRV[Title/Abstract]) |
|  | #9 | #7 OR #8 |
|  | #10 | #3 AND #6 AND #9 |
|  | #11 | #10 (Filters: English, Adult: 19+ years, Adult: 19-44 years, Aged: 65+ years, 80 and over: 80+ years, Middle Aged: 45-64 years) |
| Web of Science | #1 | (TI=(ultraso* OR sonogra* OR echo*graph*)) OR AB=(ultraso* OR sonogra* OR echo*graph*) |
|  | #2 | (TI=((enter* OR digestiv* OR oral OR sip OR nose* OR stomach* OR gastr* OR intragastr* OR nasogastr* OR intestin* OR intraintestin* OR nasointestin* OR nasoenter* OR percutaneous OR esophag* OR esophag* OR duoden* OR jejunostom* OR nasojejun*) AND (nutrition* OR feed* OR support* OR tube* OR intubat*))) OR AB=((enter* OR digestiv* OR oral OR sip OR nose* OR stomach* OR gastr* OR intragastr* OR nasogastr* OR intestin* OR intraintestin* OR nasointestin* OR nasoenter* OR percutaneous OR esophag* OR esophag* OR duoden* OR jejunostom* OR nasojejun*) AND (nutrition* OR feed* OR support* OR tube* OR intubat*)) |
|  | #3 | (TI=(EN)) OR AB=(EN) |
|  | #4 | #2 OR #3 |
|  | #5 | ((((((TI=((nutrition* OR feed* OR food OR enter* OR tube*) AND (intolera* OR toler* OR fail*))) OR TI=((reflux OR regurgitation) AND (cardio*esophageal OR esophag* OR esophag* OR gastr*))) OR TI=(aspirat* AND ("foreign bod*" OR respiratory OR accidental))) OR TI=((atoni* OR paresis OR paraly* OR stas*s OR empty* OR residu*) AND (gastric* OR stomach))) OR TI=((gastro* OR GI) AND (hemorrhage* OR hemorrhage* OR bleeding OR "blood loss"))) OR TI=((energy OR calor* OR enteral*) AND (intake* OR goal*))) OR TI=(complication* OR sequel* OR enterorrhea OR diarrh* OR flatu* OR vomit* OR emesi* OR constipation* OR "colonic Inertia" OR dyschezia OR obstipation OR GERD OR GORD OR "delayed gastric emptying" OR gastrop* OR hematochezia* OR GRV) |
|  | #6 | ((((((AB=((nutrition* OR feed* OR food OR enter* OR tube*) AND (intolera* OR toler* OR fail*))) OR AB=((reflux OR regurgitation) AND (cardio*esophageal OR esophag* OR esophag* OR gastr*))) OR AB=(aspirat* AND ("foreign bod*" OR respiratory OR accidental))) OR AB=((atoni* OR paresis OR paraly* OR stas*s OR empty* OR residu*) AND (gastric* OR stomach))) OR AB=((gastro* OR GI) AND (hemorrhage* OR hemorrhage* OR bleeding OR "blood loss"))) OR AB=((energy OR calor* OR enteral*) AND (intake* OR goal*))) OR AB=(complication* OR sequel* OR enterorrhea OR diarrh* OR flatu* OR vomit* OR emesi* OR constipation* OR "colonic Inertia" OR dyschezia OR obstipation OR GERD OR GORD OR "delayed gastric emptying" OR gastrop* OR hematochezia* OR GRV) |
|  | #7 | #5 OR #6 |
|  | #8 | #1 AND #4 AND #7 |
|  | #9 | #8 AND Article (Document Types) AND English (Languages) |
| Embase | #1 | 'echography'/exp OR 'ultrasound'/exp OR ultraso*:ti,ab,kw OR sonogra*:ti,ab,kw OR echo*graph*:ti,ab,kw |
|  | #2 | 'enteric feeding'/exp OR 'nose feeding'/exp OR 'esophagus tube'/exp OR 'jejunostomy tube'/exp OR 'nasogastric tube'/exp OR 'stomach tube'/exp |
|  | #3 | (enter*:ti,ab,kw OR digestiv*:ti,ab,kw OR oral:ti,ab,kw OR sip:ti,ab,kw OR nose*:ti,ab,kw OR stomach*:ti,ab,kw OR gastr*:ti,ab,kw OR intragastr*:ti,ab,kw OR nasogastr*:ti,ab,kw OR intestin*:ti,ab,kw OR intraintestin*:ti,ab,kw OR nasointestin*:ti,ab,kw OR nasoenter*:ti,ab,kw OR percutaneous:ti,ab,kw OR esophag*:ti,ab,kw OR esophag*:ti,ab,kw OR duoden*:ti,ab,kw OR jejunostom*:ti,ab,kw OR nasojejun*:ti,ab,kw) AND (nutrition*:ti,ab,kw OR feed*:ti,ab,kw OR support*:ti,ab,kw OR tube*:ti,ab,kw OR intubat*:ti,ab,kw) OR en:ti,ab,kw |
|  | #4 | #2 OR #3 |
|  | #5 | 'nutritional intolerance'/exp OR 'complication'/exp OR 'stomach emptying'/exp OR 'caloric intake'/exp OR 'diarrhea'/exp OR 'flatulence'/exp OR 'vomiting'/exp OR 'constipation'/exp OR 'gastroesophageal reflux'/exp OR 'foreign body aspiration'/exp OR 'stomach paresis'/exp OR 'gastrointestinal hemorrhage'/exp |
|  | #6 | (nutrition*:ti,ab,kw OR feed*:ti,ab,kw OR food:ti,ab,kw OR enter*:ti,ab,kw OR tube*:ti,ab,kw) AND (intolera*:ti,ab,kw OR toler*:ti,ab,kw OR fail*:ti,ab,kw) OR ((reflux:ti,ab,kw OR regurgitation:ti,ab,kw) AND (cardio*esophageal:ti,ab,kw OR esophag*:ti,ab,kw OR esophag*:ti,ab,kw OR gastr*:ti,ab,kw)) OR (aspirat*:ti,ab,kw AND ('foreign bod*':ti,ab,kw OR respiratory:ti,ab,kw OR accidental:ti,ab,kw)) OR ((atoni*:ti,ab,kw OR paresis:ti,ab,kw OR paraly*:ti,ab,kw OR stas*s:ti,ab,kw OR empty*:ti,ab,kw OR residu*:ti,ab,kw) AND (gastric*:ti,ab,kw OR stomach:ti,ab,kw)) OR ((gastro*:ti,ab,kw OR gi:ti,ab,kw) AND (hemorrhage*:ti,ab,kw OR hemorrhage*:ti,ab,kw OR bleeding:ti,ab,kw OR 'blood loss':ti,ab,kw)) OR ((energy:ti,ab,kw OR calor*:ti,ab,kw OR enteral*:ti,ab,kw) AND (intake*:ti,ab,kw OR goal*:ti,ab,kw)) OR complication*:ti,ab,kw OR sequel*:ti,ab,kw OR enterorrhea:ti,ab,kw OR diarrh*:ti,ab,kw OR flatu*:ti,ab,kw OR vomit*:ti,ab,kw OR emesi*:ti,ab,kw OR constipation*:ti,ab,kw OR 'colonic inertia':ti,ab,kw OR dyschezia:ti,ab,kw OR obstipation:ti,ab,kw OR gerd:ti,ab,kw OR gord:ti,ab,kw OR 'delayed gastric emptying':ti,ab,kw OR gastrop*:ti,ab,kw OR hematochezia*:ti,ab,kw OR grv:ti,ab,kw |
|  | #7 | #5 OR #6 |
|  | #8 | #1 AND #4 AND #7 |
|  | #9 | #8 AND ([adult]/lim OR [aged]/lim OR [middle aged]/lim OR [very elderly]/lim OR [young adult]/lim) AND 'Article'/it |
| CINAHL | S1 | (MM "Ultrasonography+") |
|  | S2 | (MM "Ultrasonics+") |
|  | S3 | ultraso* OR sonogra* OR echo*graph* |
|  | S4 | S1 OR S2 OR S3 |
|  | S5 | (MM "Enteral Feeding Pumps") OR (MM "Enteral Feeding (Saba CCC)") OR (MM "Enteral  Tube Feeding (Iowa NIC)") OR (MM "Feeding Tubes+") OR (MM "Enteral Nutrition") OR  (MM "Feeding Tube Care+") |
|  | S6 | (MM "Nasoenteral Tubes") OR (MM "Jejunostomy Tubes") |
|  | S7 | (enter* OR digestiv* OR oral OR sip OR nose* OR stomach* OR gastr* OR intragastr* OR nasogastr* OR intestin* OR intraintestin* OR nasointestin* OR nasoenter* OR percutaneous OR esophag* OR esophag* OR duoden* OR jejunostom* OR nasojejun*) AND (nutrition* OR feed* OR support* OR tube* OR intubat*) |
|  | S8 | EN |
|  | S9 | S5 OR S6 OR S7 OR S8 |
|  | S10 | (MM "Food Intolerance") |
|  | S11 | (MM "Energy Intake") OR (MM "Food Intake+") |
|  | S12 | (MM "Diarrhea") OR (MM "Diarrhea (NANDA)") OR (MM "Diarrhea (Saba CCC)") OR (MM  "Diarrhea Care (Saba CCC)") |
|  | S13 | (MM "Flatulence") |
|  | S14 | (MM "Vomiting+") OR (MM "Nausea and Vomiting+") OR (MM "Vomiting (Saba CCC)") |
|  | S15 | (MM "Constipation+") OR (MM "Constipation (NANDA)+") OR (MM "Constipation (Saba  CCC)+") |
|  | S16 | (MM "Gastroesophageal Reflux+") |
|  | S17 | (MM "Gastroparesis") |
|  | S18 | (MM "Gastrointestinal Hemorrhage+") |
|  | S19 | S10 OR S11 OR S12 OR S13 OR S14 OR S15 OR S16 OR S17 OR S18 |
|  | S20 | (nutrition* OR feed* OR food OR enter* OR tube*) AND (intolera* OR toler* OR fail*) |
|  | S21 | (reflux OR regurgitation) AND (cardio*esophageal OR esophag* OR esophag* OR gastr*) |
|  | S22 | aspirat* AND ("foreign bod*" OR respiratory OR accidental) |
|  | S23 | (atoni* OR paresis OR paraly* OR stas*s OR empty* OR residu*) AND (gastric* OR stomach) |
|  | S24 | (gastro* OR GI) AND (hemorrhage* OR hemorrhage* OR bleeding OR "blood loss") |
|  | S25 | (energy OR calor* OR enteral*) AND (intake* OR goal*) |
|  | S26 | (energy OR calor* OR enteral*) AND (intake* OR goal*) |
|  | S27 | complication* OR sequel* OR enterorrhea OR diarrh* OR flatu* OR vomit* OR emesi* OR  constipation* OR "colonic Inertia" OR dyschezia OR obstipation OR GERD OR GORD OR "delayed gastric emptying" OR gastrop* OR hematochezia* OR GRV |
|  | S28 | S20 OR S21 OR S22 OR S23 OR S24 OR S25 OR S26 OR S27 |
|  | S29 | S19 OR S28 |
|  | S30 | S4 AND S9 AND S29 |
| Cochrane Library | #1 | MeSH descriptor: [Ultrasonography] explode all trees |
|  | #2 | MeSH descriptor: [Ultrasonics] explode all trees |
|  | #3 | (ultraso* OR sonogra* OR echo*graph*):ti,ab,kw |
|  | #4 | #1 OR #2 OR #3 |
|  | #5 | MeSH descriptor: [Enteral Nutrition] explode all trees |
|  | #6 | ((enter* OR digestiv* OR oral OR sip OR nose* OR stomach* OR gastr* OR intragastr* OR nasogastr* OR intestin* OR intraintestin* OR nasointestin* OR  nasoenter* OR percutaneous OR esophag* OR esophag* OR duoden* OR jejunostom* OR nasojejun*) AND (nutrition* OR feed* OR support* OR tube* OR intubat*)):ti,ab,kw |
|  | #7 | (EN):ti,ab,kw |
|  | #8 | #5 OR #6 OR #7 |
|  | #9 | Any MeSH descriptor in all MeSH products and with qualifier(s): [complications - CO] |
|  | #10 | MeSH descriptor: [Gastric Emptying] explode all trees |
|  | #11 | MeSH descriptor: [Energy Intake] explode all trees |
|  | #12 | MeSH descriptor: [Diarrhea] explode all trees |
|  | #13 | MeSH descriptor: [Flatulence] explode all trees |
|  | #14 | MeSH descriptor: [Vomiting] explode all trees |
|  | #15 | MeSH descriptor: [Constipation] explode all trees MeSH |
|  | #16 | MeSH descriptor: [Gastroesophageal Reflux] explode all trees |
|  | #17 | MeSH descriptor: [Respiratory Aspiration] explode all trees |
|  | #18 | MeSH descriptor: [Gastroparesis] explode all trees |
|  | #19 | MeSH descriptor: [Gastrointestinal Hemorrhage] explode all trees |
|  | #20 | ((nutrition* OR feed* OR food OR enter* OR tube*) AND (intolera* OR toler* OR fail*)):ti,ab,kw OR ((reflux OR regurgitation) AND (cardio*esophageal OR esophag*  OR esophag* OR gastr*)):ti,ab,kw OR (aspirat* AND ((foreign NEXT bod*) OR respiratory OR accidental)):ti,ab,kw OR ((atoni* OR paresis OR paraly* OR stas*s  OR empty* OR residu*) AND (gastric* OR stomach)):ti,ab,kw OR ((gastro* OR GI) AND (hemorrhage* OR hemorrhage* OR bleeding OR "blood loss")):ti,ab,kw |
|  | #21 | ((energy OR calor* OR enteral*) AND (intake* OR goal*)):ti,ab,kw OR (complication* OR sequel* OR enterorrhea OR diarrh* OR flatu* OR vomit* OR emesi* OR  constipation* OR "colonic Inertia" OR dyschezia OR obstipation OR GERD OR GORD OR "delayed gastric emptying" OR gastrop* OR hematochezia* OR  GRV):ti,ab,kw |
|  | #22 | #9 OR #10 OR #11 OR #12 OR #13 OR #14 OR #15 OR #16 OR #17 OR #18 OR #19 OR #20 OR #21 |
|  | #23 | #4 AND #8 AND #22 |
| CNKI |  | TKA%= ( '超声' + 'B超' ) * ( '肠内营养' + '肠道营养' + '管饲' + '鼻饲' + '管喂' + '鼻胃管' + '鼻肠管' ) * ( '不耐受' + '胃肠道障碍' + '胃肠道反应' + '胃肠道症状' + '腹胀' + '肠胃胀气' + '腹满' + '腹泻' + '泄泻' + '呕吐' + '便秘' + '脾约' + '结肠无力' + '大便困难' + '反流' + '返流' + '误吸' + '胃潴留' + '胃肌轻瘫' + '胃肠道出血' + '便血' + '腹部不适' ) |
| Wanfang |  | （中英文扩展&主题词扩展）： 题名或关键词:(超声 or B超) and (肠内营养 or 肠道营养 or 管饲 or 鼻饲 or 管喂or 鼻胃管 or 鼻肠管) and (不耐受 or 胃肠道障碍 or 胃肠道反应 or 胃肠道症状 or 腹胀 or 肠胃胀气 or 腹满 or 腹泻 or 泄泻 or 呕吐 or 便秘 or 脾约 or 结肠无力 or 大便困难 or 返流 or 反流 or 误吸 or 胃潴留 or 胃肌轻瘫 or 胃肠道出血 or 便血 or 腹部不适) |
| Sinomed |  | (( "不耐受"[常用字段:智能] OR "胃肠道障碍"[常用字段:智能] OR "胃肠道反应"[常用字段:智能] OR "胃肠道症状"[常用字段:智能] OR "腹胀"[常用字段:智能] OR "肠胃胀气"[常用字段:智能] OR "腹满"[常用字段:智能] OR "腹泻"[常用字段:智能] OR "泄泻"[常用字段:智能] OR "呕吐"[常用字段:智能] OR "便秘"[常用字段:智能] OR "脾约"[常用字段:智能] OR "结肠无力"[常用字段:智能] OR "大便困难"[常用字段:智能] OR "返流"[常用字段:智能] OR "反流"[常用字段:智能] OR "误吸"[常用字段:智能] OR "胃潴留"[常用字段:智能] OR "胃肌轻瘫"[常用字段:智能] OR "胃肠道出血"[常用字段:智能] OR "便血"[常用字段:智能] OR "腹部不适"[常用字段:智能])) AND (( "超声"[常用字段:智能] OR "B超"[常用字段:智能]) AND( "肠内营养"[常用字段:智能] OR "肠道营养"[常用字段:智能] OR "管饲"[常用字段:智能] OR "鼻饲"[常用字段:智能] OR "管喂"[常用字段:智能] OR "鼻胃管"[常用字段:智能] OR "鼻肠管"[常用字段:智能])) |

**Table 2.** Acute gastrointestinal injury ultrasonography score (AGIUS score)

|  | 0 | 1 | 2 |
| --- | --- | --- | --- |
| The diameter of the intestine | < 3 cm, without changes in the intestine folds | ≥ 3 cm or changes in the intestine folds | ≥ 3 cm and changes in the intestine folds |
| The thickness of the intestine | < 3 mm, without the stratified intestinal wall | ≥ 3 mm or with stratified intestinal wall | ≥ 3 mm and with stratified intestinal wall |
| Intestinal peristalsis | 5-10/min, with transmission of intestine contents | < 5/min or > 10/min | No peristalsis, or without transmission of intestine contents |

**Table 3.** Acquisition and evaluation of GI-US predictors

| Study | GI-US predictors | Calculation formula | Time of measurement | Assessor qualifications | FI Diagnostic criteria | FI Assessment period |
| --- | --- | --- | --- | --- | --- | --- |
| Ankalagi B 2022 | GRV | 1. CSA=AP*CC*(π/4)   (2) GRV = 27.0 + 14.6 × Antral CSA (in cm^2^) − 1.28 × Age (years) | On the day of enteral feeding:   1. GRV0: before the initiation of the enteral feed； 2. GRV1, GRV2, GRV3, and GRV4: every hour for the first 4 hours | (1) Radiologist (initial 35 scans)  (2) Principal investigator (subsequent) | FI was defined clinically by symptoms such as abdominal pain, discomfort, abdominal distention, regurgitation, or vomiting. | On the day of EN |
| Chen B 2024 | SMA-PSV  SMA-EDV  SMA-PI  SMA-RI | Not Reported | Days 1, 3, 7 of EN | Single physician | Gastrointestinal symptoms such as constipation, diarrhea, abdominal distension and gastric retention occur during EN and result in the suspension or reduction of EN, making it impossible to achieve the target calorie intake within 72 h. | Days 1, 3, 7 of EN |
| Chen C 2020 | GCSA | Free-tracing technique (mean of 3 measurements) | Daily during EN | Critical care ultrasound-trained physician | During the implementation of EN, when the nutrients are continuously infused for ≥ 6 hours, the detected value of the patient's gastric residual volume is > 250 mL or uncomfortable symptoms such as abdominal distension, diarrhea, vomiting or reflux appear. | EN period |
| El Khoury D 2023 | GCSA | Free-tracing tool | Not reported | Trained operators | Defined by composite criteria: regurgitation, discontinuation of EN, nasogastric tube aspiration, <20% of previous day's calorie intake due to inability to receive EN. | Within 24 h postmeasurement |
| Fu H 2024 | AMI | 1. AMI = ACF × ACA 2. ACF: Number of antral contractions every 2 min 3. ACA= (S diastolic - S contractile)/S diastolic | Pre-EN;  Days 1-3 post-EN | Not Reported | FI can be diagnosed when one or more of the following three items occur: (1) Adverse gastrointestinal reactions, including vomiting, reflux, abdominal distension, diarrhea, constipation, GRV ≥ 500 mL/24 h, and digestive system bleeding; (2) After 72 h, the energy supply of 83.68 kJ·kg⁻¹·d⁻¹ still cannot be achieved; (3) Interruption of EN caused by reasons other than medical staff. | Days 1-3 post-EN initiation |
| Gao T 2019 | Intestinal Diameter,  Intestinal Peristalsis,  Intestinal Wall Thickness,  Alterations in Intestinal Folds, Intestinal Wall Stratification,  AGIUS Score | Not reported | Daily (first week post-admission) | Two attending physicians | Defined as the interruption of EN because of a gastrointestinal issue (severe abdominal distention, diarrhea, vomiting, gastric residual volume ≥ 300 mL in 6 h, or subjective discomfort) . | 6 h/12 h post-EN initiation |
| Lai J 2022 | GCSA,  Colonic Diameter, Colonic Peristalsis | GCSA: antral edge tracing | Pre-EN; 24 h, 72 h, 120 h post-EN | Certified ultrasound researchers | EN failure: Feeding intolerance occurs.When GRV is 500  ml within 6  h and gastrointestinal symptoms such as vomiting, diarrhea, abdominal pain and distension occur, gastric motility drugs were allowed within 72 h of EN, and when the target feeding was not achieved after 120 h of EN, this was judged as failure. | EN period |
| Li T 2024 | GCSA,  GRV | 1. GCSA = (coronal diameter × sagittal diameter × π)/4, 2. GRV = 27 + 14.6 × CSA − 1.28 × age | 6-hourly for 5 days (≥3×/day) | Not Reported | 1. High GRV: GRV>250 mL after 4 h continuous drip or>500 mL after 24 h, (2) Abdominal distension: subjective feeling of abdominal distension, or abdominal pressure>15 mmHg, (3) Diarrhea: >3 times/d, and the total amount is > 250 mL/d, (4) Vomiting, reflux. | Days 1-3 postadmission |
| Onuk S, 2023 | GCSA | GCSA= (coronal diameter × sagittal diameter × π)/4 | Baseline (10–15 min post-EN);  Days 3, 5after EN initiation | ICU physician (ultrasound-certified) | GIDS score (grades 1–4). | Days 1-5 post-EN initiation |
| Pérez-Calatayud AA 2022 | GCSA,  CGV,  ΔGCSA,  GRV | 1. ΔGCSA = (A−B)/A × 100 2. CGV = 27.0 + 14.6 × GCSA −1.28 × age | Pre-UMAT; 2,5,10,20,30,60 min post-EN | Senior ICU physicians (validated experience) | FI defined as a continued rise of Intra-Abdominal Pressure (IAP) or a change in the AGI grade, abdominal distension, gastric residue >500 ml after 5 h of enteral nutrition, and ileus did not resolve after 5 h of EN. | First 48 h of EN |
| Sharma R 2023 | US-guided GRV | 1. GCSA: free-tracing; 2. GRV = 27.0 + 14.6 × CSA −1.28 × age | Twice daily (morning/evening) during EN | Radiologist (initial scans) | FI defined by observing symptoms of vomiting, flatulence, bowel movement, abdominal distension with pain, and radiological evaluations. | Daily |
| Taskin G 2021 | GCSA | GCSA= 3.142 × (dAP × dCC) ∕ 4 | Twice daily until EN cessation | Experienced intensivist | Gastric contents were slowly aspirated with 50 ml syringes for at least 10 min until stomach contents could no longer be aspirated. The aspirated GRV was noted in milliliters. | Twice daily after ultrasound assessment |
| Wang L 2022 | Echodensity of Gastric Antrum Wall | Not reported | Daily prefeeding (first ICU week) | Experienced physician | FI was defined as the occurrence of the following symptoms resulting in forced interruption of EN: vomiting or regurgitation, diarrhea, ileus, and mesenteric ischemia/perforation. | Days 1-7 ICU admission |
| Xiang M 2024 | GRV | 1. GCSA = π × (AP diameter × CC diameter)/4, 2. GRV = 27 + 14.6 × GCSA − 1.28 × age | Day 3 of EN | Experienced sonographers | References were provided | First EN week |
| Yu G 2023 | TGIU parameters,  GCSA,  AGIUS | 1. TGIU parameters：   including GCSA and AGIUS   1. GCSA = π × (AP diameter × CC diameter)/4, 2. GRV = 27 + 14.6 × GCSA − 1.28 × age | 6-hourly during first EN week | Certified operators | The definition of FI included (1) high GRV, GRV>250 mL after 4 h continuous infusion; (2) gastrointestinal (GI)symptoms (vomiting, diarrhea, and/or abdominal distension) > 3 times/d, and (3) intra-abdominal pressure (IAP) > 15 mmHg (1 mmHg ¼ 0.133 kPa). | Days 1,3,5,7 of EN |
| Zou T 2019 | GCSA | GCSA = (coronal diameter × sagittal diameter × π)/4 | 4 h post-EN (Days 1-3) | Certified ICU technicians | FI defined by symptoms: ①High GRV: GRV >250 mL after 4 h of continuous drip; ② Abdominal distension: subjective feeling of abdominal distension, or abdominal pressure >15 mmHg (1 mmHg = 0.133 kPa); ③ Diarrhea: >3 times/d, and the total amount of ＞250 mL/d; ④ Vomiting, reflux. | Days 1-3 post-EN initiation |

Note: FI: Feeding Intolerance; EN: Enteral Nutrition; GI: Gastrointestinal; CSA: Cross-Sectional Area; GRV: Gastric Residual Volume; AMI: Antral Motility Index; SMA: Superior Mesenteric Artery Velocities; PSV: Peak Systolic Velocity; EDV: End-Diastolic Velocity; US-guided GRV: Ultrasound-Guided Gastric Residual Volume; AGIUS Score: Acute Gastrointestinal Injury Ultrasonography Score; TGIU: Transabdominal Gastrointestinal Ultrasonography (TGIU); GIDS: Gastrointestinal Dysfunction Score​​

**Table 4.**  Evaluation Results of the GCSA-FI Predicted Performance

| First author, Year | GI-US predictors | Sample Size for Prediction | Measurement position | Cutoff | Sensitivity | Specificity | AUC |
| --- | --- | --- | --- | --- | --- | --- | --- |
| Chen C 2020 | GCSA (semi-reclining) | 43 | Semi-reclining | ≥ 3.917 cm^2^ | 0.920 | 0.692 | 0.815 |
|  | GCSA (supine) |  | Supine | ≥ 3.395 cm^2^ | 0.696 | 0.923 | 0.833 |
|  | GCSA (RLD) |  | RLD | ≥ 4.402 cm^2^ | 0.923 | 0.714 | 0.849 |
| El Khoury D 2023 | GCSA | 44 | half-seated (45°) | 7.20 cm^2^ | 0.910 | 0.810 | 0.861 |
| Lai J 2022 | GCSA | 105 | RLD/HOB 30-45° | ≤9cm^2^ | 0.835 | 0.811 | 0.896 |
| Li T 2024 | GCSA | 30 | Supine | 7.835 cm^2^ | 0.882 | 0.718 | 0.828 |
| Onuk S 2023 | GCSA | 39 | HOB 30° | >4.48 cm^2^ | 0.727 | 0.772 | 0.768 |
| Pérez-Calatayud, AA 2022 | Basal GCSA | 61 | HOB 30° |  |  |  |  |
|  | ∆GCSA 2 min |  |  |  |  |  |  |
|  | ∆GCSA 5 min |  |  |  |  |  |  |
|  | ∆GCSA 10 min |  |  |  |  |  |  |
|  | ∆GCSA 20 min |  |  |  |  |  |  |
|  | ∆GCSA 30 min |  |  |  |  |  |  |
|  | ∆GCSA 60 min |  |  | 52% | 0.500 | 0.889 | / |
| Taskin G 2021 | GCSA | 56 | Not Reported | 9.22 cm2 | 1.000 | 0.913 | 0.969 |
| Yu G 2023 | GCSA | 91 | Supine/RLD | ≥6.0 cm^2^ | 0.860 | 0.794 | 0.763 |
| Zou T 2019 | GCSA | 150 | Supine | 7.92cm^2^ | 0.727 | 0.755 | 0.699 |

Note: RLD: Right lateral decubitus, HOB 30-45°: Head of the bed elevated 30-4530–45 degrees, HOB 30°: Head of the bed elevated 30 degrees; GCSA: Gastric Cross-sectional Area

**Table 5.** Sensitivity analysis of pooled diagnostic metrics via the leave-one-out approach

| Omitted_Study | Sensitivity | Specificity | AUC | DOR | PLR | NLR |
| --- | --- | --- | --- | --- | --- | --- |
| Chen C 2020 | 0.82 (0.75-0.87) | 0.77 (0.72-0.81) | 0.86 (0.76-0.89) | 15.04 (9.29-24.36) | 3.56 (2.91-4.37) | 0.24 (0.17-0.33) |
| El Khoury D 2023 | 0.82 (0.75-0.87) | 0.76 (0.71-0.80) | 0.85(0.76-0.89) | 14.46 (8.94-23.41) | 3.43 (2.81-4.18) | 0.24 (0.17-0.33) |
| Lai J 2022 | 0.83 (0.75-0.89) | 0.75 (0.70-0.80) | 0.77 (0.75-0.89) | 14.74 (8.67-25.05) | 3.34 (2.72-4.10) | 0.23 (0.16-0.33) |
| Li T 2024 | 0.82 (0.75-0.87) | 0.78 (0.73-0.82) | 0.86 (0.76-0.89) | 15.56 (9.44-25.63) | 3.68 (2.95-4.58) | 0.24 (0.17-0.33) |
| Onuk S 2023 | 0.84 (0.77-0.89) | 0.77 (0.72-0.81) | 0.87 (0.77-0.90) | 17.04 (10.30-28.21) | 3.58 (2.94-4.36) | 0.21 (0.15-0.30) |
| Yu G 2023 | 0.81 (0.72-0.87) | 0.76 (0.72-0.81) | 0.85 (0.75-0.88) | 13.48 (7.84-23.18) | 3.40 (2.77-4.19) | 0.25 (0.17-0.37) |
| Zou T 2019 | 0.84 (0.77-0.89) | 0.77 (0.71-0.82) | 0.88(0.76-0.90) | 17.60 (10.31-30.06) | 3.67 (2.90-4.65) | 0.21 (0.15-0.30) |

**​**

**Fig. 1** Forest plot of leave-one-out sensitivity analysis (sensitivity and specificity)​

​


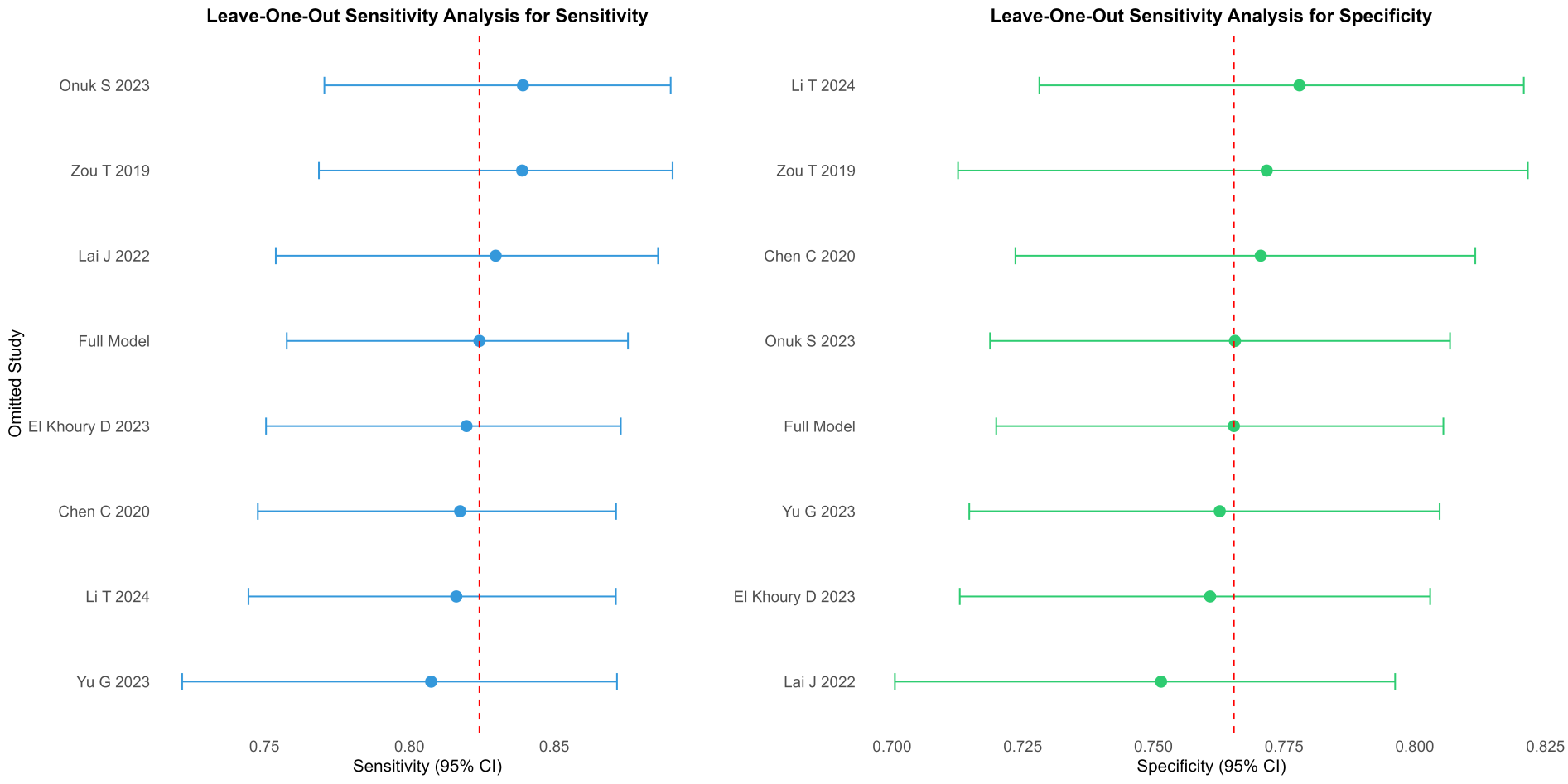


**Fig. 2** Forest plot of leave–one-out sensitivity analysis (positive and negative likelihood ratios)​**​**


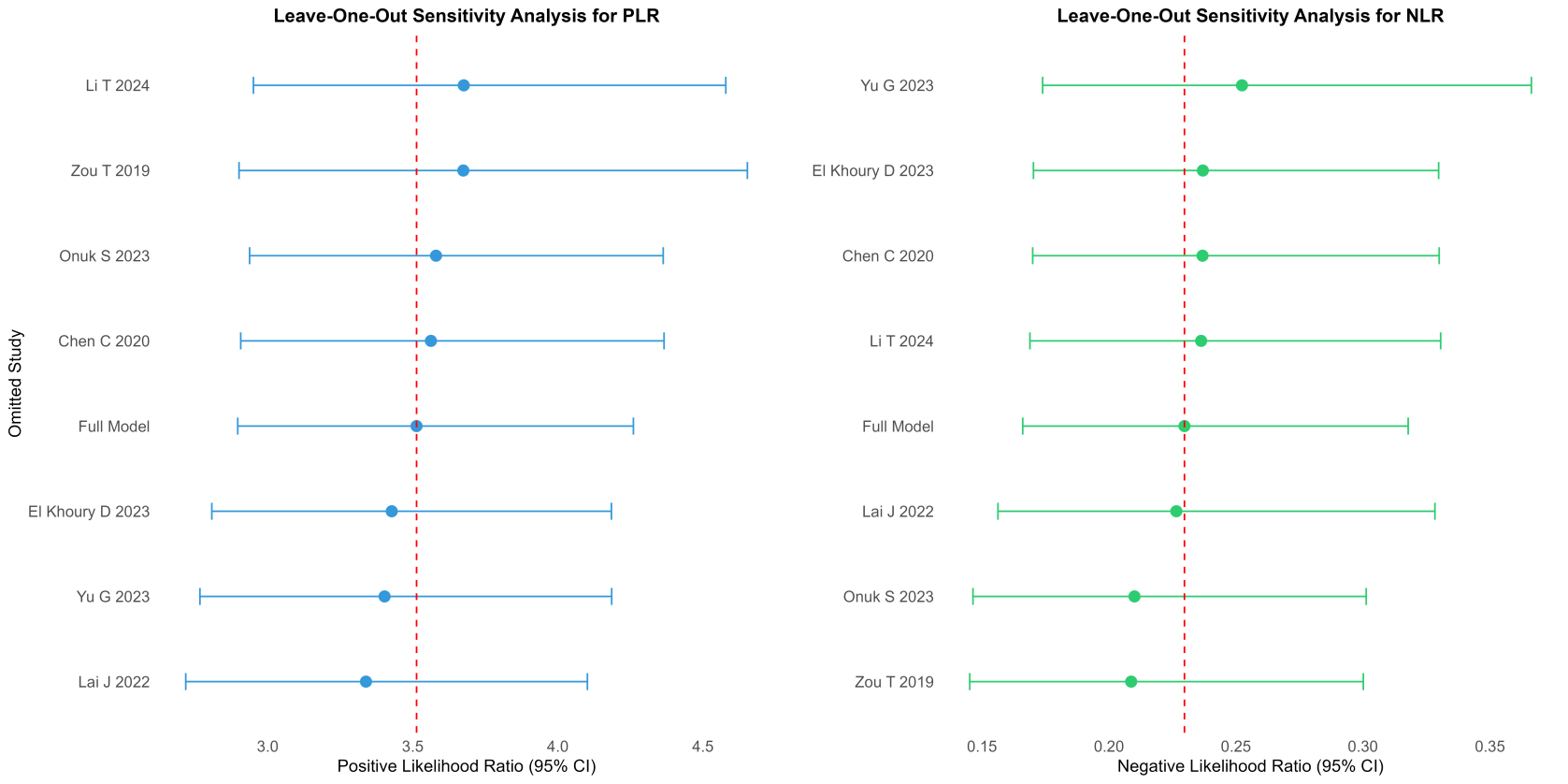


**
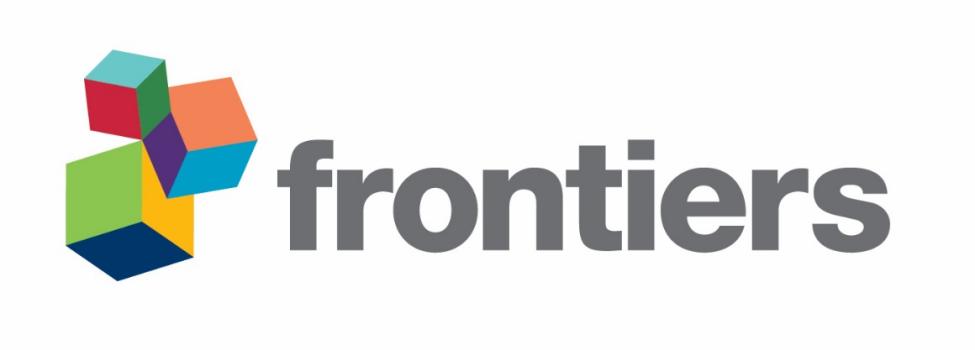
**
